# Transient Sepsis-Induced Cardiomyopathy in the Setting of Alcohol Withdrawal

**DOI:** 10.7759/cureus.102588

**Published:** 2026-01-29

**Authors:** Jonathan Van Name

**Affiliations:** 1 Internal Medicine, University of Florida College of Medicine, Gainesville, USA

**Keywords:** alcohol withdrawal, bacteremia, cardiogenic shock, gram-negative bacteremia, sepsis, undifferentiated shock

## Abstract

Sepsis-induced cardiomyopathy (SICM) is a reversible myocardial dysfunction that occurs in the setting of severe sepsis and septic shock, often complicating the management of patients with underlying cardiovascular disease. We report the case of a 62-year-old man with heart failure with improved ejection fraction (HFimpEF), paroxysmal atrial fibrillation, and severe alcohol use disorder who developed septic shock due to *Enterococcus faecalis* bacteremia, complicated by multiorgan failure and transient worsening of cardiac function. Myocardial dysfunction was attributed to SICM based on the temporal association with septic shock, elevated lactate and vasopressor requirement, new right ventricular dilation and hemodynamic compromise on transthoracic echocardiography, and subsequent recovery with sepsis resolution. Alternative etiologies were considered and excluded, including acute coronary syndrome, recurrent pulmonary embolism, and persistent tachycardia-mediated cardiomyopathy. Echocardiographic abnormalities emerged contemporaneously with bacteremia and shock onset and improved in parallel with hemodynamic stabilization and clearance of infection. Despite profound circulatory collapse, myocardial function recovered with supportive care, with clinical and echocardiographic improvement observed within days of shock resolution, reinforcing the reversible nature of SICM rather than alternative etiologies (e.g., acute coronary syndrome, recurrent pulmonary embolism, etc.). This case highlights the diagnostic challenges of SICM, particularly in patients with pre-existing heart failure and atrial fibrillation, and underscores its transient, reversible nature.

## Introduction

Sepsis remains a leading cause of morbidity and mortality worldwide and is frequently complicated by cardiovascular dysfunction. Among the most clinically significant manifestations is sepsis-induced cardiomyopathy (SICM), a reversible myocardial dysfunction that occurs in the setting of severe sepsis or septic shock. Contemporary echocardiographic studies estimate that SICM affects approximately 20-50% of patients with septic shock, depending on diagnostic criteria and timing of assessment, underscoring its clinical relevance in the intensive care setting [1]. Despite this prevalence, SICM remains underrecognized, in part because its presentation is heterogeneous and often overlaps with other causes of acute cardiac dysfunction in critically ill patients.

The pathophysiology of SICM is multifactorial and incompletely understood. Proposed mechanisms include inflammatory cytokine-mediated myocardial depression, nitric oxide-dependent alterations in contractility, mitochondrial dysfunction, impaired calcium handling, autonomic dysregulation, and microcirculatory abnormalities leading to myocardial stunning [2]. Importantly, these processes occur in the absence of irreversible myocyte necrosis, distinguishing SICM from ischemic cardiomyopathy and accounting for its characteristic reversibility. Advances in bedside echocardiography have expanded the conceptual framework of SICM beyond isolated reductions in left ventricular ejection fraction (LVEF), revealing frequent involvement of diastolic dysfunction and right ventricular impairment.

Patients with heart failure with improved ejection fraction (HFimpEF) represent a diagnostically challenging subgroup in whom SICM may be particularly difficult to identify. In such patients, a preserved or near-normal LVEF during sepsis may falsely reassure clinicians, as baseline expectations for systolic function differ from those in patients without prior cardiomyopathy. Acute septic myocardial dysfunction may instead manifest as impaired cardiac reserve, ventriculoarterial uncoupling, or right ventricular failure rather than overt reduction in LVEF. Consequently, reliance on LVEF alone may obscure clinically significant myocardial dysfunction and delay recognition of SICM in patients with HFimpEF.

Distinguishing SICM from other transient cardiomyopathies encountered in critical illness is essential. Stress-induced (Takotsubo) cardiomyopathy typically presents with regional wall motion abnormalities triggered by catecholamine surges, while tachycardia-mediated cardiomyopathy develops over days to weeks of sustained rapid ventricular rates and improves only after prolonged rhythm or rate control [3]. In contrast, SICM is characterized by global myocardial dysfunction temporally linked to sepsis and shock, with recovery occurring in parallel with resolution of the inflammatory state. Failure to differentiate these entities may lead to inappropriate diagnostic testing or premature escalation to advanced heart failure therapies [4,5].

Although prior literature has emphasized the diagnostic difficulty of SICM in sepsis, fewer reports have examined its presentation in patients with HFimpEF complicated by concurrent alcohol withdrawal syndrome and atrial fibrillation - a clinical scenario in which sympathetic activation, arrhythmia burden, and sepsis-related inflammation converge. This case addresses this gap by illustrating how SICM may present despite preserved LVEF, how alternative etiologies can be systematically excluded, and how longitudinal assessment clarifies reversibility. By contextualizing SICM within this complex physiologic overlap, this report aims to refine clinical recognition and inform management strategies in similarly high-risk patients.

## Case presentation

**Figure 1:**
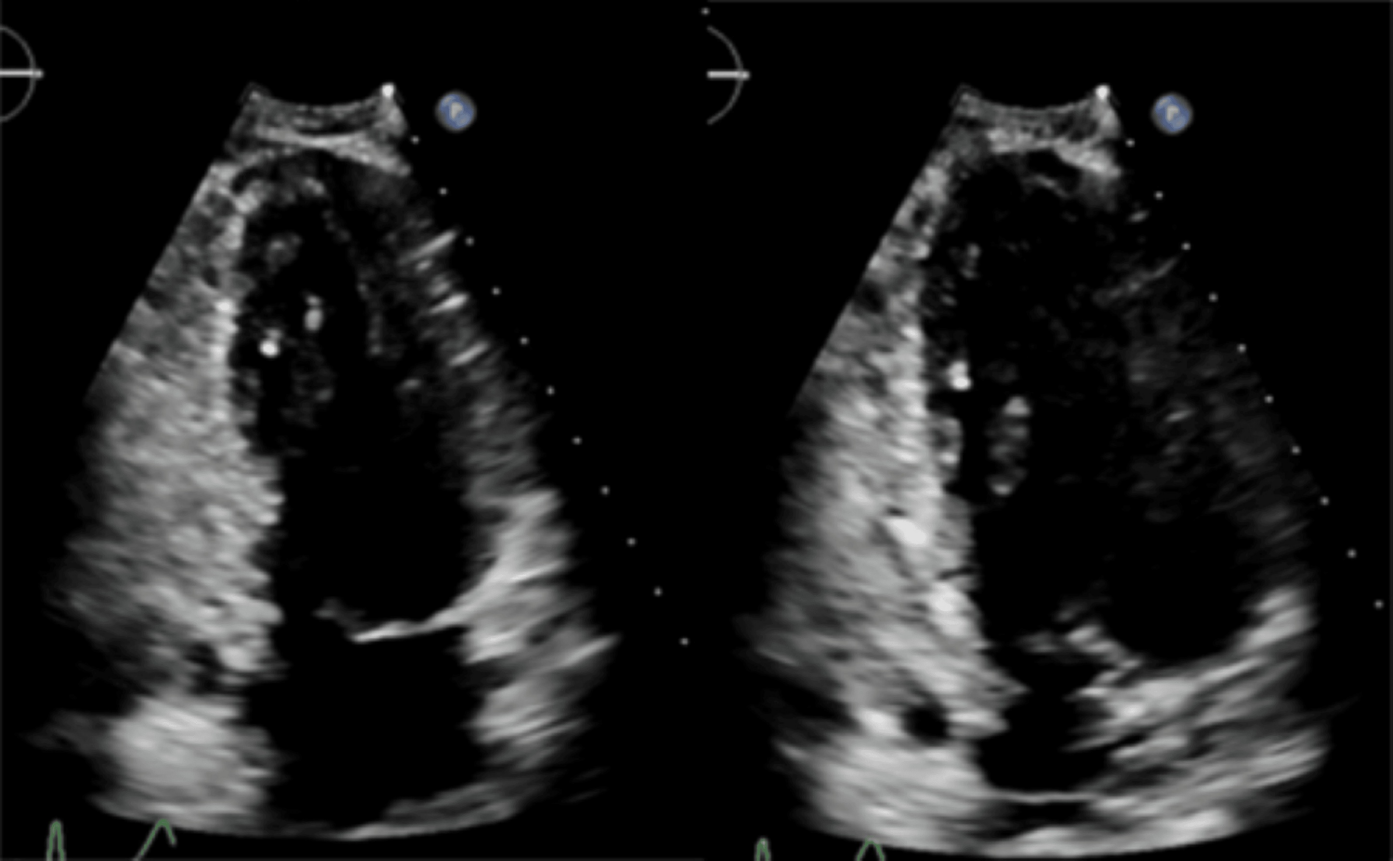
Transthoracic echocardiogram showing improved ejection fraction after resolution of sepsis

A 62-year-old man with a history of severe alcohol use disorder complicated by prior withdrawal seizures and delirium tremens, paroxysmal atrial fibrillation (status post cardioversion in January 2024, on apixaban), recent pulmonary embolism, hypertension, hypothyroidism, and HFimpEF (most recent LVEF 50-54% in November 2025) presented on January 5, 2026 with dizziness, weakness, tremors, and gastrointestinal symptoms consistent with alcohol withdrawal. His baseline creatinine was 0.7 mg/dL, and there was no known history of chronic liver disease, chronic kidney disease, or structural valvular abnormalities. Transthoracic echocardiogram images obtained after improvement in heart function are shown below in Figure 1.

On presentation, the patient was tachycardic with atrial fibrillation and rapid ventricular response, hypotensive, and hypoxic, requiring supplemental oxygen. Initial laboratory evaluation demonstrated a high anion gap metabolic acidosis, hyperkalemia, elevated blood urea nitrogen, and mild hyperbilirubinemia. Given the absence of fever, leukocytosis, or a clear infectious source at admission, and in the context of severe alcohol withdrawal with autonomic instability, empiric antibiotics were initially deferred in favor of close monitoring and supportive care. Early hypotension was attributed to volume depletion, arrhythmia-related hemodynamic compromise, and sedative effects rather than overt sepsis.

Shortly after admission, escalating agitation and autonomic instability necessitated treatment with benzodiazepines and phenobarbital as part of a withdrawal management protocol. Cumulative dosing included repeated intravenous benzodiazepine boluses followed by a phenobarbital loading regimen and taper. Following sedation, the patient developed worsening hypotension and decreased responsiveness, prompting endotracheal intubation for airway protection and initiation of vasopressor support with norepinephrine. Mean arterial pressures during this period were persistently below 65 mmHg prior to vasopressor initiation. Central venous access was obtained later in the course; invasive filling pressures were not available during the initial shock episode.

The patient stabilized over the subsequent 24 hours and was extubated on hospital day two, with vasopressors weaned off. However, his hospital course was complicated by recurrent atrial fibrillation with rapid ventricular response requiring escalating beta-blockade, fluctuating mental status attributed to ongoing alcohol withdrawal, and progressive metabolic derangements. Initial blood cultures remained negative, and empiric antimicrobial therapy was discontinued after no infection was identified during the early phase of hospitalization. On hospital day five, the patient acutely decompensated with mixed septic and cardiogenic shock, evidenced by hypotension, elevated lactate, and severe biventricular dysfunction, necessitating re-initiation of vasopressor support with norepinephrine. Serum lactate rose to 8.3 mmol/L, accompanied by severe transaminase elevation (aspartate aminotransferase (AST) peaking at approximately 17,000 U/L and alanine aminotransferase (ALT) at 4,000 U/L), acute kidney injury with creatinine increasing to 1.54 mg/dL, hyperkalemia to 6.8 mmol/L, and evolving coagulopathy, consistent with shock-related multiorgan failure. The overall hemodynamic profile, marked by vasopressor dependence, rising lactate, and end-organ hypoperfusion in the setting of arrhythmia and underlying cardiomyopathy, suggested a mixed septic and cardiogenic shock physiology. 

Evaluation for alternative causes of shock was undertaken. Serial electrocardiograms demonstrated atrial fibrillation without ischemic ST-segment changes, and cardiac biomarkers did not show a pattern consistent with acute coronary syndrome. Given the patient’s history of pulmonary embolism, recurrent thromboembolism was considered; however, there was no evidence of acute right heart strain on imaging beyond what was attributed to sepsis-related hemodynamic stress, and clinical suspicion for recurrent pulmonary embolism remained low.

Transthoracic echocardiography performed during this decompensation demonstrated preserved left ventricular systolic function but new right ventricular dilation with elevated estimated pulmonary pressures. No regional wall motion abnormalities were identified. Earlier echocardiographic images from the initial admission were not obtained due to clinical instability and prioritization of airway and hemodynamic management; this limitation was mitigated by comparison with a well-documented baseline echocardiogram from November 2025 and repeat imaging during the shock state and recovery. A timeline of the hemodynamic, laboratory, and cardiac findings from the hospitalization is summarized in Table 1. 

**Table 1:** Timeline of Hemodynamic, Laboratory, and Cardiac Findings During Hospitalization AF = atrial fibrillation, RVR = rapid ventricular response, TTE = transthoracic echocardiogram, RV = right ventricle, LVEF = left ventricular ejection fraction, ALT = alanine aminotransferase, AST = aspartate aminotransferase, AKI = acute kidney injury

Hospital Day	Key Clinical Events	Vasopressor Support	Peak Lactate (mmol/L)	Creatinine (mg/dL)	AST / ALT (U/L)	Cardiac Rhythm	Cardiac Imaging / Hemodynamic Findings
Day 1	Presentation with alcohol withdrawal syndrome, AF with RVR, acute hypoxic respiratory failure; intubation	Yes	~3–4	0.7	Mildly elevated	AF with RVR	Prior TTE (1 month prior to admission): LVEF 50–54%
Day 2	Extubation; vasopressors weaned; ongoing alcohol withdrawal	No	<2	0.8	Mild	AF	No acute cardiac imaging
Day 4	Recurrent hallucinations; tachycardia requiring escalation of beta-blockade	No	<2	~1.0	Mild ↑	AF with RVR	—
Day 5	Acute decompensation with shock, altered mental status, oliguria	Yes	8.3	1.54	~17,000 / 4,000	AF with RVR	TTE: Markedly reduced LVEF (15-20%), new RV dilation, elevated pulmonary pressures
Day 6	Septic shock confirmed; Enterococcus faecalis bacteremia; multiorgan failure	Yes	↓	3–4	Markedly elevated	AF	Mixed septic–cardiogenic physiology
Day 7–8	Hemodynamic stabilization; vasopressors discontinued >48 h	No	Normalizing	Peak 4.9–5.6	Improving	Rate-controlled AF	Clinical improvement
Day 10	Recovery phase; polyuric AKI; improving hepatic function	No	Normal	↓	↓	Controlled	Resolution of shock and myocardial dysfunction. Repeat TTE normal EF.

Broad-spectrum antibiotics were reinitiated, and blood cultures subsequently grew Enterococcus faecalis, with polymerase chain reaction confirming the organism and no vancomycin resistance genes detected. A focused source evaluation was undertaken, including assessment for urinary, gastrointestinal, and intravascular sources, though no definitive nidus was identified. The bacteremia was managed with targeted antimicrobial therapy in consultation with infectious diseases specialists.

With appropriate antimicrobial treatment, vasopressor support, and careful volume management, the patient’s hemodynamics gradually improved. Vasopressors were discontinued after more than 48 hours of stability, lactate levels normalized, and end-organ function recovered. Renal failure progressed transiently to a polyuric phase, with creatinine peaking above 5 mg/dL before gradual improvement. Hepatic transaminases and coagulation parameters steadily normalized. Serial clinical assessments and follow-up echocardiography demonstrated resolution of shock and improvement in cardiac function, consistent with the reversible course characteristic of SICM.

## Discussion

This case demonstrates key features of SICM while highlighting the diagnostic complexity posed by concurrent atrial fibrillation with rapid ventricular response and alcohol withdrawal syndrome. Differentiating SICM from other transient cardiomyopathies was central to clinical decision-making. Tachycardia-mediated cardiomyopathy typically develops after sustained periods of uncontrolled tachyarrhythmia over days to weeks and improves gradually following durable rate or rhythm control [6]. In contrast, this patient experienced abrupt hemodynamic collapse temporally linked to septic shock, with myocardial dysfunction emerging and resolving in parallel with infection control and shock resolution rather than with arrhythmia suppression alone. Similarly, stress-induced (Takotsubo) cardiomyopathy was considered but was felt to be unlikely given the absence of regional wall motion abnormalities and the presence of global hemodynamic compromise rather than a classic apical or mid-ventricular pattern.

Cardiac biomarkers further supported a diagnosis of septic myocardial stunning rather than ischemic injury. Serial electrocardiograms did not demonstrate ischemic ST-segment changes, and cardiac biomarker trends were not consistent with acute coronary syndrome. While mild troponin elevation is commonly observed in sepsis and reflects myocardial strain rather than plaque rupture, no dynamic rise-and-fall pattern suggestive of ischemia was observed in this case. Natriuretic peptide levels, when elevated in sepsis, may reflect ventricular wall stress and volume shifts rather than intrinsic systolic failure, limiting their specificity [7]. Taken together, the biomarker profile and electrocardiographic findings supported a non-ischemic, reversible myocardial process.

The temporal relationship between shock onset, echocardiographic abnormalities, and antimicrobial initiation further reinforces the diagnosis of SICM. Echocardiographic evidence of cardiac dysfunction, particularly right ventricular dilation and elevated pulmonary pressures, coincided with the onset of septic shock and rising lactate levels. Improvement in hemodynamics and cardiac performance occurred after initiation of targeted antimicrobial therapy and resolution of bacteremia, rather than immediately following rate control or withdrawal management [8]. This sequence underscores the central role of sepsis-related inflammation and circulatory derangements in driving myocardial dysfunction.

Importantly, reliance on LVEF alone may be misleading in the setting of sepsis. Reduced systemic vascular resistance and altered afterload conditions can preserve or even artifactually normalize LVEF despite impaired myocardial contractile reserve. In patients with HFimpEF, baseline expectations for systolic function further complicate interpretation. In this case, preserved LVEF masked clinically significant myocardial dysfunction manifested by vasopressor dependence, elevated lactate, right ventricular failure, and end-organ hypoperfusion. These findings emphasize the need to interpret echocardiographic parameters within the broader hemodynamic and clinical context rather than as isolated measures.

The absence of advanced invasive hemodynamic monitoring, such as pulmonary artery catheterization, represents an important limitation in precisely characterizing shock physiology. Without direct measurements of cardiac output, filling pressures, and pulmonary vascular resistance, differentiation between septic, cardiogenic, and mixed shock relied on clinical assessment, laboratory markers, echocardiography, and response to therapy [9]. However, this approach reflects real-world practice in many intensive care units, where pulmonary artery catheters are used selectively. Serial bedside assessments and the patient’s rapid improvement with sepsis-directed therapy provided pragmatic confirmation of a predominantly sepsis-driven, reversible myocardial process.

Finally, this case highlights how alcohol withdrawal syndrome may act as an amplifier rather than a primary driver of myocardial dysfunction. Sympathetic activation, catecholamine excess, and arrhythmia burden likely reduced cardiovascular reserve and lowered the threshold for decompensation during sepsis [10]. Recognition of this interaction is essential, as failure to disentangle these overlapping processes may obscure the diagnosis of SICM and delay appropriate management. Together, these considerations reinforce the importance of a comprehensive, temporal, and physiology-based approach to diagnosing SICM in complex critically ill patients.

## Conclusions

SICM is an important and often underrecognized contributor to shock and transient worsening of heart failure in patients with severe sepsis. This case highlights the heterogeneous presentation of SICM, including preserved left ventricular systolic function with concomitant right ventricular dysfunction, and reinforces that myocardial impairment in sepsis may be clinically significant despite a seemingly reassuring LVEF. Recognition of this reversible process is essential to avoid misclassification of shock physiology and unnecessary escalation to advanced heart failure therapies.

Alcohol withdrawal syndrome in this patient was temporally associated with cardiovascular instability and likely functioned as a compounding physiologic stressor rather than a primary cause of myocardial dysfunction. The coexistence of alcohol withdrawal, atrial fibrillation with rapid ventricular response, and septic shock illustrates the inherent diagnostic uncertainty in mixed shock states and underscores the importance of longitudinal assessment. This case adds to existing SICM literature by demonstrating how baseline HFimpEF can obscure sepsis-related myocardial dysfunction and by emphasizing the diagnostic value of integrating clinical timing, echocardiography, and response to antimicrobial therapy.

From a practical standpoint, clinicians should consider repeat echocardiographic evaluation after hemodynamic stabilization and reassessment of chronic heart failure therapies as sepsis resolves. Future studies and case aggregation focusing on SICM in patients with alcohol use disorder may further clarify how withdrawal-related autonomic stress influences myocardial dysfunction and recovery, ultimately improving diagnostic precision and patient management.
